# Molecular Mechanism of Strict Substrate Specificity of an Extradiol Dioxygenase, DesB, Derived from *Sphingobium* sp. SYK-6

**DOI:** 10.1371/journal.pone.0092249

**Published:** 2014-03-21

**Authors:** Keisuke Sugimoto, Miki Senda, Daisuke Kasai, Masao Fukuda, Eiji Masai, Toshiya Senda

**Affiliations:** 1 Department of Materials Chemistry, Asahikawa National College of Technology, Asahikawa, Hokkaido, Japan; 2 Structural Biology Research Center, Photon Factory, Institute of Materials Structure Science, High Energy Accelerator Research Organization (KEK), Tsukuba, Ibaraki, Japan; 3 Department of Bioengineering, Nagaoka University of Technology, Nagaoka, Niigata, Japan; NCI-Frederick, United States of America

## Abstract

DesB, which is derived from *Sphingobium* sp. SYK-6, is a type II extradiol dioxygenase that catalyzes a ring opening reaction of gallate. While typical extradiol dioxygenases show broad substrate specificity, DesB has strict substrate specificity for gallate. The substrate specificity of DesB seems to be required for the efficient growth of *S.* sp. SYK-6 using lignin-derived aromatic compounds. Since direct coordination of hydroxyl groups of the substrate to the non-heme iron in the active site is a critical step for the catalytic reaction of the extradiol dioxygenases, the mechanism of the substrate recognition and coordination of DesB was analyzed by biochemical and crystallographic methods. Our study demonstrated that the direct coordination between the non-heme iron and hydroxyl groups of the substrate requires a large shift of the Fe (II) ion in the active site. Mutational analysis revealed that His124 and His192 in the active site are essential to the catalytic reaction of DesB. His124, which interacts with OH (4) of the bound gallate, seems to contribute to proper positioning of the substrate in the active site. His192, which is located close to OH (3) of the gallate, is likely to serve as the catalytic base. Glu377’ interacts with OH (5) of the gallate and seems to play a critical role in the substrate specificity. Our biochemical and structural study showed the substrate recognition and catalytic mechanisms of DesB.

## Introduction

Clarification of the mechanisms by which bacteria adapt to a given environment is one of the critical challenges in the field of evolutionary biology. It has been considered that protein molecules in the cell change their characteristics to adapt to the environment, as observed in thermophilic bacteria [1]. Recently an adaptation of a bacterial enzyme under specific culture conditions was also reported [2]. However, the details of the adaptation strategy of enzymes are not well understood. In order to analyze the enzyme evolution, we have investigated a bacterium, *Sphingobium* sp. strain SYK-6 [3], isolated from pulping waste liquor. This bacterium can grow with various lignin-derived aromatic compounds as sole carbon sources, because it has evolved a degradation pathway for these aromatic compounds.

**Figure 1:**
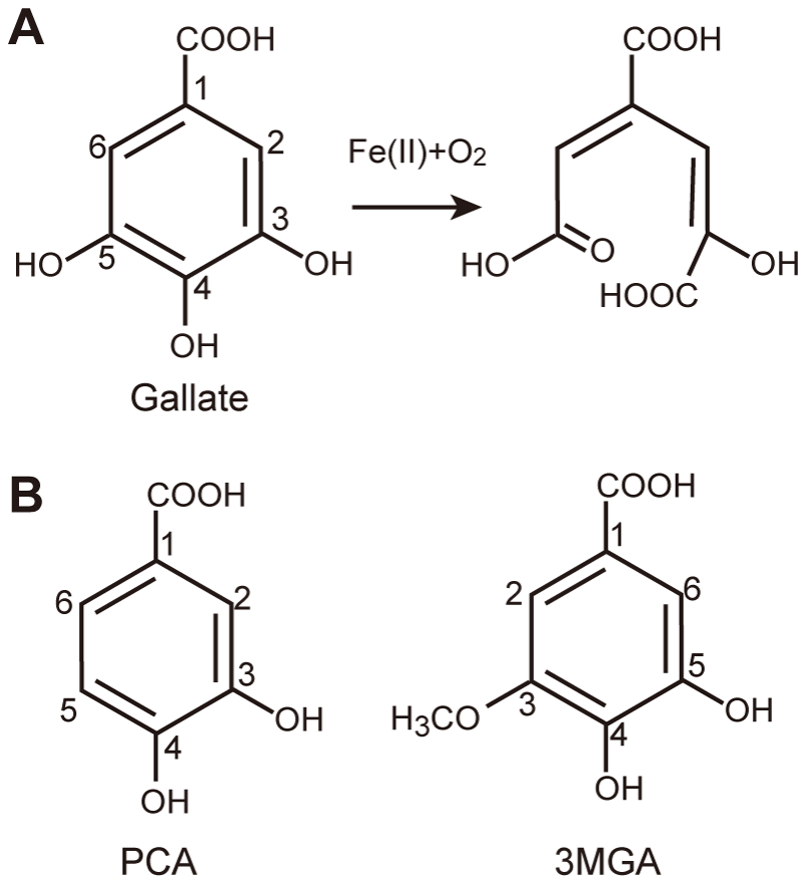
Catalytic reaction of DesB. (**A**) The chemical structure of gallate and the catalytic reaction of DesB. (**B**) The chemical structures of PCA and 3MGA.

*S.* sp. SYK-6 is the best characterized of the bacteria that can catabolize lignin-derived aromatic compounds such as syringate and vanillate [4]. Since lignin is one of the most abundant carbon sources on earth, mineralization of lignins is a critical step in the terrestrial carbon cycle. *S.* sp. SYK-6 converts syringate and vanillate into three catecholic compounds, gallate, protocatechuate (PCA), and 3-*O*-methylgallate (3MGA), which are structurally similar to one another (**Figures 1A, B**). The three catecholic compounds are degraded by three evolutionarily related type II extradiol dioxygenases–DesB, LigAB, and DesZ–in *S.* sp. SYK-6 [4]–[6]. Two of these three enzymes, LigAB and DesZ, show broad substrate specificity like other extradiol dioxygenases [6], [7]. However, the enzyme activities of these two enzymes for gallate are insufficient to metabolize syringate. Accumulation of gallate, which is a metabolite of syringate, in the cell inhibits the cell growth of a *desB* deletion-mutant strain. On the other hand, DesB exhibits not only a strict substrate specificity but also high enzyme activity for gallate [6] (**Table S1**). This enzyme property is critical for the growth of *S.* sp. SYK-6 in the presence of lignin-derived aromatic compounds. However, it is unknown how the bacterium has evolved the unique extradiol dioxygenase that is highly specific for gallate.

While extradiol dioxygenases can be categorized into two major groups, types I and II [8], their catalytic mechanisms seem to be essentially the same due to their convergent evolution [9]. In both types of extradiol dioxygenases, the catalytic reaction initiates from a direct coordination of the two hydroxyl groups of a catecholic substrate to the non-heme Fe (II) ion in the active site. The coordination geometry of the substrate is asymmetric in both types of extradiol dioxygenases; equatorial and axial hydroxyl-group ligands show short and long coordination distances, respectively [9]–[13]. Furthermore, all extradiol dioxygenases possess a catalytic base adjacent to the axial hydroxyl group of the substrate [10]–[16]. In addition, biochemical analyses of extradiol dioxygenases have demonstrated that extradiol dioxygenases typically show broad substrate specificity, while each of them shows a specific preference among possible substrates. Although there have been some structure-based studies on the mechanism of the substrate specificity of extradiol dioxygenases, details of the substrate-specificity mechanism remain elusive.

In this study, we analyzed the mechanism of the substrate recognition of DesB on the basis of the crystal structures of reaction intermediates. Our biochemical and structural analyses suggested that DesB shares the typical catalytic mechanism of the estradiol dioxygenases. Interestingly, a large shift of the Fe (II) ion was observed in the DesB-gallate complex. The shift of the Fe (II) ion, which seems to be critical to the catalytic reaction, is likely to be induced by the substrate binding at the productive binding site and to cause the strict substrate specificity of DesB.

## Materials and Methods

### Crystal structure analysis of DesB

DesB was crystallized as described previously [17]. Briefly, DesB was overexpressed using *Escherichia coli* and purified to near homogeneity. The purified DesB was concentrated to about 14–30 mg/ml and crystallized through mixing with crystallization solution (25% (w/v) PEG8000, 0.1 M sodium acetate trihydrate, 0.1 M HEPES-NaOH pH 7.75). In order to prevent the oxidation of the Fe (II) ion in the active site, all crystallization procedures were performed under anaerobic conditions. Since the crystallization solution contained 25% (w/v) PEG8000, no cryo-protection was required. The obtained crystals were frozen using liquid nitrogen. Diffraction data of DesB crystals were collected at PF of KEK (Tsukuba, Japan) (**Table 1**).

**Table 1:** Crystallographic summary of DesB (I).

	*Anaerobic substrate-free*	*Anaerobic gallate complex*	*Aerobic gallate complex*	*Anaerobic gallate complex*	*Anaerobic PCA complex*
**Mutation**	Wild type	Wild type	Wild type	His124Phe	Wild type
**Data collection**					
Beam Line	PF BL-5A	PF BL-5A	PF BL-5A	PF-AR NE3A	BL-5A
Space group	*P*2_1_	*C*222_1_	*P*2_1_	*P*2_1_	*P*2_1_
**Cell dimensions**					
*a*	56.42	123.52	57.37	57.35	57.83
*b*	64.92	159.18	64.86	65.52	61.01
*c* (Å)	116.25	104.50	117.66	117.88	117.55
α, β, γ (°)	90, 94.83, 90	90, 90, 90	90, 96.24, 90	90, 96.93, 90	90, 97.84, 90
**Resolution (Å)**	56.6 – 2.25	53.2 – 2.40	56.7 – 2.10	58.5 – 2.10	54.1 – 2.40
(Highest shell)	(2.37 – 2.25)	(2.53 – 2.40)	(2.21 – 2.10)	(2.20 – 2.10)	(2.53 – 2.40)
*R*_merge_	0.080 (0.333)	0.115 (0.608)	0.044 (0.309)	0.067 (0.194)	0.037 (0.296)
*I*/σ*I*	10.35 (3.25)	12.06 (3.18)	15.34 (3.43)	9.82 (4.59)	14.7 (3.26)
Completeness (%)	99.3 (99.6)	99.9 (100.0)	97.5 (96.9)	98.3 (97.4)	94.4 (85.0)
Redundancy	1.8 (1.8)	4.6 (4.6)	2.2 (2.3)	2.2 (2.1)	2.0 (1.9)
**Refinement**					
Resolution (Å)	56.6 – 2.25	53.2 – 2.40	55.0 – 2.10	58.0 – 2.10	54.0 – 2.40
No. reflections	37,880	38,540	46,934	47,891	29,790
*R*_work_/*R*_free_	0.1909/0.2392	0.1849/0.2388	0.2103/0.2562	0.1920/0.2425	0.2170/0.2760
**No. atoms**					
Protein	6,458	6,585	6,447	6,506	6,378
Ligand/ion	2	28	2	26	13
Water	282	165	200	543	42
**B-factors**					
Protein	22.1	28.5	34.6	22.5	65.5†
Ligand/ion	20.9	25.5	31.8	28.1	28.5
Water	24.5	26.0	30.8	27.2	21.9
**R.M.S. deviations**					
Bond lengths (Å)	0.010	0.012	0.012	0.011	0.011
Bond angles (°)	1.210	1.293	1.368	1.210	1.189
**PDB ID**	3WR8	3WR9	3WRA	3WRB	3WRC

The highest resolution shell is shown in parenthesis.

WT: wild type.

H124F: His124Phe

† Protomer A shows substantially higher averaged B-factor (89.2 Å^2^) than that of protomer B (42.9 Å^2^). PCA was observed only in protomer B.

The crystal structure of DesB was determined by the molecular replacement (MR) method using the program MOLREP [18]. The coordinates of LigAB (PDB ID: 1BOU), which shows 36% amino acid sequence identity to DesB, were utilized as a search model for the MR analysis. The molecular model of DesB was constructed using the program Coot [19] and refined using the program REFMAC5 [20]. The Ramachandran plot of the refined crystal structure (PDB ID: 3WR8), which was calculated using the program PROCHECK in the CCP4 program suite [21], showed that there are no residues that have φ ˜ψ angles in the disallowed regions. As a result, 0.4% (3 residues), 8.1% (56 residues) and 91.4% (630 residues) of residues were found in the generously allowed, allowed and favored regions of the φ ˜ψ angles, respectively.

### Crystal structure analysis of the anaerobic DesB-gallate and DesB-PCA complexes

Crystals of the DesB-gallate (the wild type and the His124Phe mutant) and DesB-PCA complexes were prepared under anaerobic conditions using an anaerobic chamber that was specially designed for anaerobic crystallographic experiments [22]. DesB was crystallized under anaerobic conditions and soaked in artificial mother liquor solution (25% (w/v) PEG8000, 0.1 M sodium acetate trihydrate, 0.1 M HEPES-NaOH pH 7.75) containing 10 mM gallate or 10 mM PCA for two hours. The soaked crystals were frozen in the anaerobic chamber using liquid nitrogen. Diffraction data of these crystals were collected at BL-5A or PF-AR NE3A in PF (Tsukuba, Japan). The crystal structures of these complexes were determined by the MR method with the program MOLREP [18] using the refined DesB structure as a search model. The crystal structures were refined using the programs REFMAC5 [20] (**Tables 1** **and** **2**).

**Table 2:** Crystallographic summary of DesB (II).

	*Anaerobic gallate complex*	*Anaerobic gallate complex*	*Anaerobic gallate complex*	*Anaerobic gallate complex*
		*(co-crystal)*	*(No Fe shift)*	*(No Fe shift)*
**Mutation**	**Wild type**	**Wild type**	**Wild type**	**Wild type**
**Data collection**				
Beam Line	PF BL-5A	PF BL-17A	PF BL-5A	PF BL-5A
Space group	*P*2_1_	*P*2_1_	*P*2_1_	*P*2_1_
**Cell dimensions**				
*a*	57.44	58.10	58.35	58.30
*b*	60.77	64.18	64.02	63.91
*c* (Å)	117.99	117.99	118.48	118.30
α, β, γ (°)	90, 98.58, 90	90, 97.18, 90	90,97.14,90	90,96.98,90
**Resolution (Å)**	56.8 – 2.70	56.3 – 2.50	19.7 – 2.50	20.0 – 2.40
(Highest shell)	(2.85 – 2.70)	(2.64 – 2.50)	(2.63 – 2.50)	(2.52 – 2.40)
*R*_merge_	0.044 (0.393)	0.042 (0.313)	0.043 (0.354)	0.037 (0.254)
*I*/σ*I*	16.01 (3.09)	18.37 (3.32)	14.46 (3.28)	14.42 (3.06)
Completeness (%)	98.2 (98.5)	99.8 (99.8)	90.9 (89.4)	85.9 (66.4)
Redundancy	2.3 (2.4)	2.5 (2.5)	2.3 (2.3)	1.8 (1.7)
**Refinement**				
Resolution (Å)	20.0 – 2.70	56.3 – 2.50	19.7 – 2.50	20.0 – 2.40
No. reflections	20,862	30,057	28,045	31,674
*R*_work_/*R*_free_	0.2329/0.3144	0.2254/0.2957	0.2313/0.3130	0.2214/0.2946
**No. atoms**				
Protein	6,449	6,288	6,449	6,449
Ligand/ion	14	14	14	14
Water	2	1	0	0
**B-factors**				
Protein	61.7†	42.2†	60.01†	51.93†
Ligand/ion	32.9	25.5	44.26	38.41
Water	30.2	14.1	-	
**R.M.S. deviations**				
Bond lengths (Å)	0.011	0.009	0.010	0.009
Bond angles (°)	1.319	1.235	1.335	1.314
**PDB ID**	3WKU	3WPM	3WR3	3WR4

The highest resolution shell is shown in parenthesis.

† Protomer A shows substantially higher averaged B-factor than that of protomer B. (**Table 1**). Gallate was observed only in protomer B.

The Ramachandran plot of the anaerobic DesB-gallate complex (PDB ID: 3WR9) showed that there are no residues that have φ ˜ψ angles in the disallowed regions, and 0.7% (5 residues), 7.5% (53 residues) and 91.8% (648 residues) of residues were found in the generously allowed, allowed and favored regions of the φ ˜ψ angles, respectively. As for the DesB-PCA complex (PDB ID: 3WRC), there are no residues that have φ ˜ψ angles in the disallowed regions, and 0.3% (2 residues), 10.7% (72 residues) and 89.1% (602 residues) of residues were found in the generously allowed, allowed and favored regions of the φ ˜ψ angles, respectively.

### Crystal structure analysis of the DesB-gallate complex exposed to the aerobic conditions (the aerobic DesB-gallate complex)

Crystals of the DesB-gallate complex were prepared as described above. The crystals were soaked for 2 hours, then taken out from the anaerobic chamber and exposed to an aerobic atmosphere. After 30-min of incubation under aerobic conditions, the crystals were frozen using liquid nitrogen. Diffraction data were collected at BL-5A of the PF in KEK (Tsukuba, Japan). The crystal structure of the aerobic DesB-gallate complex was determined using the MR method with the program MOLREP [18]. Crystallographic refinement was carried out using the program REFMAC5 [20] (**Table 1**). The Ramachandran plot of the anaerobic DesB-gallate complex (PDB ID: 3WRA) showed that there are no residues that have φ ˜ψ angles in the disallowed regions, and 0.7% (5 residues), 8.0% (55 residues) and 91.3% (628 residues) of residues were found in the generously allowed, allowed and favored regions of the φ ˜ψ angles, respectively.

### Crystal structure analysis of the DesB-gallate complex by co-crystallization

To obtain further information on the DesB-gallate complex, DesB was co-crystallized with gallate under anaerobic conditions. DesB was concentrated to about 30 mg/ml and mixed with 5 mM of gallate at a 1∶1 volume ratio to prepare the DesB-gallate complex. Then the DesB-gallate complex was crystallized through mixing with crystallization solution (26% (w/v) PEG8000, 0.1 M sodium acetate trihydrate, 0.1 M HEPES-NaOH pH 7.75) under anaerobic conditions. The obtained crystals were cryo-protected by soaking in 25% (w/v) PEG8000, 0.1 M sodium acetate, 0.1 M HEPES pH 7.75 and 10 mM gallate for 20 min and then frozen using liquid nitrogen. Diffraction data of DesB crystals were collected at BL-17A in PF of KEK (Tsukuba, Japan). The crystal structures of these complexes were determined by the MR method with the program MOLREP [18] using the refined DesB structure as a search model. The crystal structures were refined using the program PHENIX [23] (PDB ID: 3WPM, **Table 2**).

### Biochemical analysis of the mutants and their CD spectra

The His124Phe and His192Phe mutants of DesB were prepared using the PCR method. In order to confirm the proper folding of these mutants, circular dichroism (CD) spectra were measured using a CD spectrometer (JASCO, Japan).

### Measurement of enzyme activity

The wild type and mutant forms of DesB were purified to near homogeneity as described previously [17]. Before the measurement of enzyme activity, DesB was reactivated through mixing of the reactivation solution and apo-form DesB (apo-DesB), which was prepared by treatment with a chelating reagent. The purified DesB was incubated for 12 hours at 4°C in buffer A (0.1 M NaCl, 20 mM Tris-HCl, 10% glycerol, pH 8.0) containing EDTA with a 20-fold excess (molar ratio) of DesB. Then the apo-DesB was concentrated to 50 mg/ml in buffer B (20 mM Tris-HCl, 10% glycerol, pH 8.0) by centrifugation with Centriplus YM-10 (Millipore). The concentrated apo-DesB was frozen by liquid nitrogen and stored at −80°C. Apo-DesB was reactivated under anaerobic conditions. First, apo-DesB was diluted to 10 mg/ml using buffer B. Then, the diluted apo-DesB (100 μL) was mixed with 100 μL of the reactivation solution (50 mM MOPS, 5% (v/v) acetone, 4 mM ascorbate, 2 mM FeCl_2_, pH 7.5) at 4°C. The reactivated enzyme typically kept its enzyme activity for about 4 days under the present conditions. Enzyme activity was analyzed in air-saturated 50 mM Tris-HCl (pH 7.5) at 30°C by measuring the decrease of the substrate (gallate) concentration. The concentration of gallate was determined from absorption at 265 nm with a molar absorption coefficient of 8,133 M^−1^cm^−1^. Since the product of the enzyme reaction also has absorption at 265 nm, the net amount of decreased gallate was calculated with consideration of the absorption of the product at 265 nm (ε_265nm_  =  1,616 M^−1^cm^−1^). Specific activities were calculated by considering the auto-degradation of gallate under aerobic conditions.

It is of note that the conditions of the enzyme assay in this study were different from those reported earlier [6]. Since extradiol dioxygenases readily lose their activity due to dissociation of the Fe (II) ion from the active site, it is critical to reactivate the enzyme to obtain stable and reliable results. We have therefore established a method to reactivate DesB, and this reactivation method was utilized in this study. DesB was reactivated before the enzyme assay as described above. This reactivation procedure significantly improved the enzyme activity of DesB. The difference of the reaction conditions may also cause the difference of the specific activities.

The enzymatic activities of these mutants were also measured with crude extract of cultured *E. coli* based on the oxygen consumption as described earlier [6]. The oxygen consumptions of the His124Phe and His192Phe mutants were below the detectable level.

### Structural comparison and molecular graphics

The crystal structures were superimposed using the program LSQKAB in the CCP4 program suite [21]. All molecular graphics in the present manuscript were prepared using the program PyMOL (Schrödinger, Cambridge, MA).

### Data deposition

The atomic coordinates and structure factors of the anaerobic substrate-free (3WR8), anaerobic gallate complex (in *C*222_1_) (3WR9), anaerobic gallate complex (in *P*2_1_) (3WKU, 3WR3, 3WR4), aerobic gallate complex (3WRA), anaerobic gallate complex of the His124Phe mutant (3WRB), anaerobic PCA complex (3WRC), and anaerobically co-crystallized gallate complex (3WPM) forms are deposited in the Protein Data Bank with the accession numbers shown in parentheses (**Tables 1** **and** **2**).

## Results

### Crystal structures of DesB and its anaerobic substrate complex

**Figure 2:**
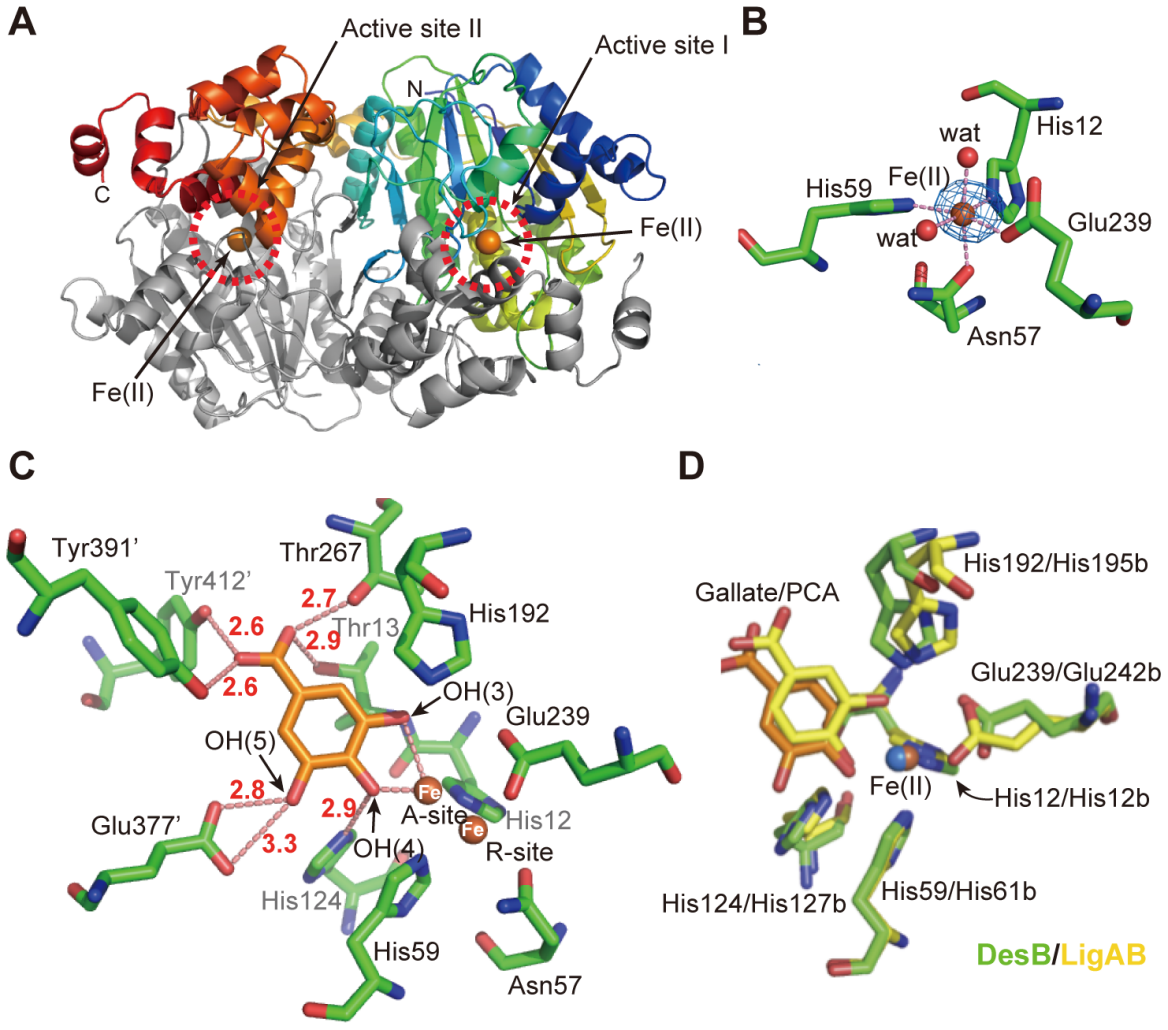
Structure of DesB. (**A**) The crystal structure of the DesB dimer. The asymmetric unit of the crystal contains the whole dimer. Subunit A is shown in rainbow colours along the polypeptide chain from the N (blue) to the C (red) terminus. Subunit B is shown in light grey. (**B**) The Fe (II) coordination sphere of DesB and an anomalous difference Fourier map (contour level of 5.0 σ). (**C**) The active site of the anaerobic DesB-gallate complex. Carbon atoms of bound gallate are shown in orange. Hydrogen bonds are indicated by pink dotted lines with distances in Å units. (**D**) Comparison of the coordination spheres between DesB (carbon atoms in green) and LigAB (carbon atoms in yellow) in the substrate (PCA) complex forms [9]. Carbon atoms of gallate are shown in orange.

The crystal structure of DesB was determined at 2.25 Å resolution by the molecular replacement method using the coordinates of LigAB (PDB ID: 1BOU) as a search model (**Figure 2A** **,** **Table 1** **, Figure S1**). While LigAB is an α_2_β_2_-type tetramer [9], DesB is a β_2_-type homo-dimer. The α and β subunits of LigAB correspond to residues 285−418 (C-terminal domain) and 2−284 (N-terminal domain) of DesB, respectively. The crystal structure of DesB shows that DesB is a swap dimer, in which the N-terminal domain forms a protomer with the C-terminal domain of the adjacent subunit in the dimer (**Figure 2A**). Protomer A (B) was determined to be composed of residues 1−300 of subunit A (B) and residues 301-C-terminal of subunit B (A). The active sites of DesB, each of which is located at the interface of the two subunits, contain a non-heme Fe (II) ion. The Fe (II) ion coordinates four protein ligands, His12 (2.2 Å), Asn57 (2.3 Å), His59 (2.3 Å), and Glu239 (2.1 Å), and two water molecules (2.2 Å and 2.4 Å) (the averaged coordination distances of the two active sites are given in parentheses) (**Figure 2B**). The structure of the coordination sphere in DesB was essentially the same as that of LigAB.

The B-factors of the Fe (II) ions are comparable to those of ligand atoms; the averaged B-factor of the Fe (II) ions is 21.0 Å^2^ and that of the ligand atoms is 18.0 Å^2^ (**Table S2**), suggesting that most of the Fe (II) sites of DesB molecules in the crystal are occupied by Fe (II) ions. Although the X-ray diffraction data of the DesB crystal in the substrate-free form were collected at a wavelength of 1.0000 Å, the anomalous difference Fourier map showed a clear density of more than 5 σ at the Fe (II) site (**Figure 2B**). The position of the Fe (II) ion could therefore be examined by using anomalous difference Fourier maps.

In order to analyze the mechanism of the substrate specificity of DesB, the crystal structure of the anaerobic DesB-gallate complex (in the space group of *C*222_1_) was determined. The crystal structure of the substrate complex showed that gallate is bound to the active site through several hydrogen bonds (**Figure 2C**). Since the B-factors of the bound gallate, of which occupancy was set to 1.0 in the crystallographic refinement, were comparable to those of the surrounding atoms, the occupancy of the bound gallate seemed to be nearly 1.0 (**Table S2**). The hydrogen bonds with Tyr391’ and Tyr412’ seemed to stabilize the loop region (residues 410’−417’), the electron densities of which were not observed in the substrate-free form (the prime at the end of the residue number shows that the residue belongs to the adjacent subunit in the DesB dimer). Two hydroxyl groups of gallate, OH (3) and OH (4), directly coordinate the Fe (II) ion as observed in other extradiol dioxygenases [9−13], . The Fe (II)-OH (4) distance (1.9 Å) is significantly shorter than the Fe (II)-OH (3) distance (2.6 Å) (**Table S3**).

His192, which is a conserved residue in type II dioxygenases, is located adjacent to OH (3) of the substrate, which is an axial ligand of the Fe (II) ion (**Figure 2C**). The OH (3)-Fe (II) distance suggests that OH (3) is not deprotonated as observed in other extradiol dioxygenases [9]–[13]. The His192Phe mutant, which is likely to preserve the tertiary structure as demonstrated by the CD spectrum (**Figure S2**), shows non-detectable enzyme activity (less than 0.01 U/mg), while the wild-type DesB shows a specific activity of 116.1±0.5 U/mg. The catalytic mechanism of extradiol dioxygenases so far proposed suggests that His192 plays a role as a catalytic base of this enzyme [11]–[16]. It is of note that the His192Phe mutant may not be able to bind the Fe (II) ion properly at the active site. However, since His192 is located approximately 6 Å from the Fe (II) site, a negative effect on the Fe (II) binding seems to be unlikely.

His124, which is also conserved in type II dioxygenases, forms a hydrogen bond with OH (4) of the substrate, which is an equatorial ligand of the Fe (II) ion. The coordination distance between OH (4) and Fe (II) suggests that OH (4) is deprotonated as observed in other extradiol dioxygenases [9−13], [15], [16], . Since the His124Phe mutant shows no detectable enzyme activity (less than 0.01 U/mg), His124 is also indispensable to the enzyme activity for DesB. Furthermore, crystallographic analysis of this mutant showed that the His124Phe mutation is unlikely to affect the Fe (II) binding at the active site (see below, **Table S2**). Earlier studies of extradiol dioxygenases suggested that His124 contributes to the deprotonation of the substrate by forming a hydrogen bond with OH (4).

The active-site architecture of the DesB-substrate complex is similar to those of the types I and II extradiol dioxygenases, suggesting that the observed binding structure of gallate is likely to be a productive one (**Figure 2D** **, Figure S3**) [9], [25], [26]. The gallate-binding site in the DesB-gallate complex is therefore designated as the productive binding site of DesB.

### Fe-ion shift upon substrate binding

**Figure 3:**
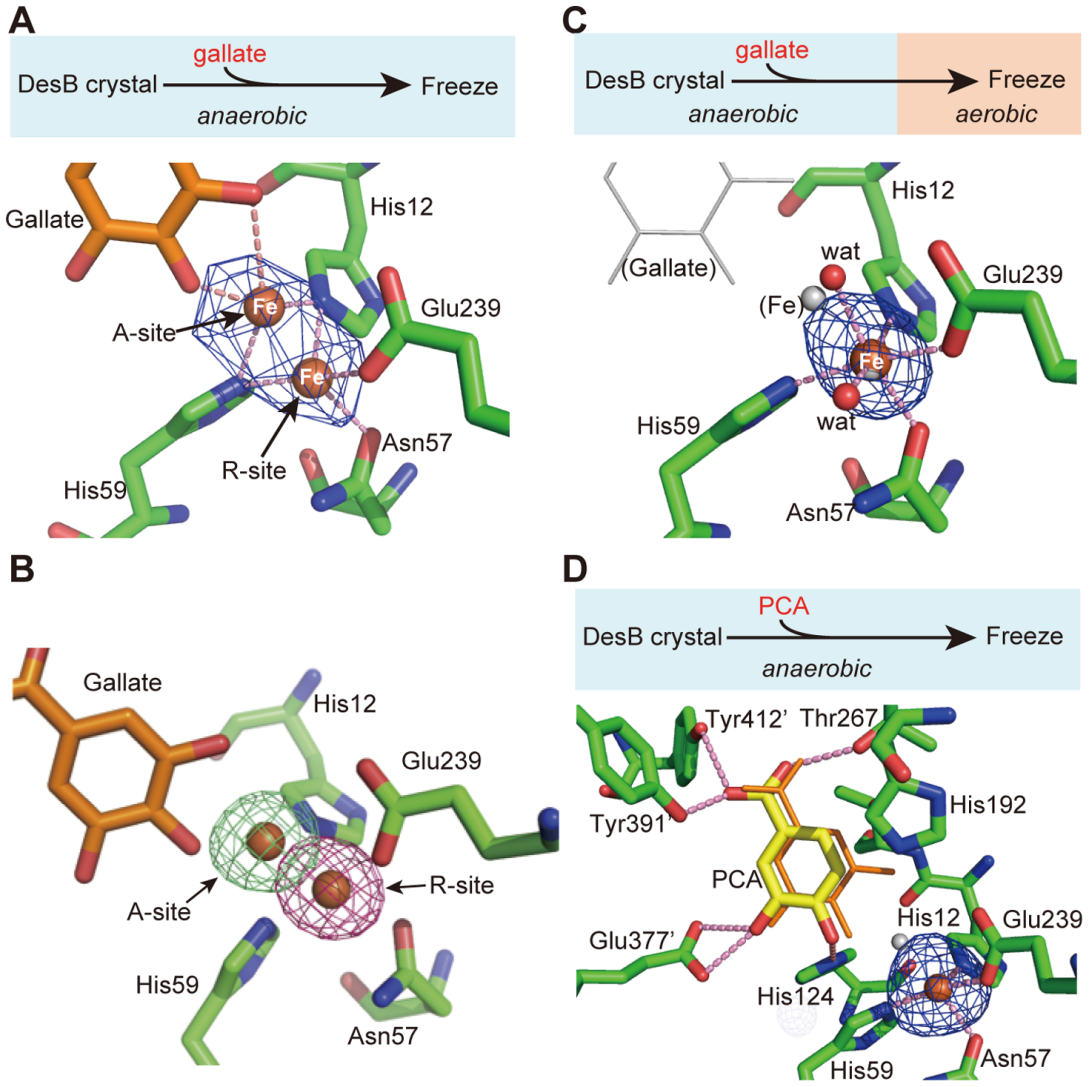
Active site structures of the DesB-gallate and DesB-PCA complexes. (**A**) Crystal preparation scheme of the anaerobic DesB-gallate complex (upper panel) and alternative conformations of the Fe (II) ion observed in the anaerobic DesB-gallate complex (lower panel). An anomalous difference Fourier density (blue) is contoured at the 5.0 σ level. (**B**) Omit maps for A- and R-site Fe (II) ions (5 σ.. The green and red densities are omit maps for the Fe (II) ions at the A- and R-sites, respectively. (**C**) Crystal preparation scheme of the aerobic DesB-gallate complex (upper panel) and an anomalous difference Fourier map (5.0 σ) of the aerobic DesB-gallate complex (lower panel). Thin white sticks and small white spheres indicate the positions of gallate and the Fe ion found in the anaerobic DesB-gallate complex, respectively. (**D**) Crystal preparation scheme of the anaerobic DesB-PCA complex (upper panel) and its active site structure with an anomalous difference Fourier map (5.0 σ) (lower panel). Carbon atoms of PCA are shown in yellow. The thin stick model in orange indicates the position of gallate in the anaerobic DesB-gallate complex.

Despite the similarity of the active site structure to those of other extradiol dioxygenases, one extraordinary feature was found in DesB. In the anaerobic DesB-gallate structure, the Fe (II) ion in the active site adopts alternative conformations (R- and A-sites) (**Figure 2C**). First, an anomalous difference Fourier map contoured at the 5 σ level shows a long ellipsoidal electron density that covers possible alternative positions of the Fe (II) ion (**Figure 3A**). Second, omit maps for each Fe position also support the alternative conformations of the Fe (II) ion (**Figure 3B**). On the basis of these results, we concluded the alternative conformations of the Fe (II) ion. The occupancy of the Fe (II) ion in each position was not refined, because the resolution of the DesB-gallate complex was not sufficient. The occupancy of each Fe (II) ion of the alternative conformations was set to 0.5 (**Table S2**).

The R-site is the same as the Fe (II) site of the substrate-free form. On the other hand, the Fe (II) ion at the A-site is located at a distance of approximately 2 Å from the R-site and coordinates OH (3) and OH (4) of gallate. Due to the large shift of the Fe (II) ion, the Fe (II) ion at the A-site loses coordination with Asn57 and Glu239 in both active sites I and II of the DesB dimer **(** **Figure 2C** **, Table S3**). Since the conformations of the side chains of His12 and His59 have ambiguities due to the moderate resolution of the crystal structure, the precise coordination structure of the Fe (II) ion in the active site of the DesB-gallate complex remains elusive. The side chains of His12 and His59 may interact with the Fe (II) ion at the A-site (**Table S3**). Notably, a similar shift of the Fe (II) ion was observed in the LigAB-PCA complex. The Fe (II) shift in LigAB was less than that in DesB and the Fe (II) coordination structure was retained after the Fe (II) shift [9]. Details of the coordination structure of the DesB-gallate complex should be analyzed by high-resolution crystal structure and spectroscopic methods in solution.

The crystal of the DesB-gallate complex described above belongs to the space group *C*222_1_. However, only one crystal of the space group *C*222_1_ was obtained in this study. Therefore, the crystal that belonged to the space group *P*2_1_ was used for further analysis (**Table 2**). The *P*2_1_ crystals of the DesB-gallate complex, however, were of poor quality; the electron density around the active site of protomer A was disordered and electron density for gallate could not be observed in protomer A. Since no significant disorder was found in the substrate-free form of DesB crystals, the disordered protomer A seemed to arise from the conformational change associated with the substrate binding under anaerobic conditions. Therefore, *m*Fo-*D*Fc omit maps for the Fe (II) ion were examined in protomer B. We collected four diffraction datasets of the DesB-gallate complex from *P*2_1_ crystals. While two of these datasets showed no clear alternative conformations of the Fe (II) ion, the other two suggested alternative conformations of the Fe (II) ion (**Figure S4**).

The two crystal structures of the DesB-gallate complex without alternative conformations of the Fe (II) ion, however, showed a weak but significant *m*Fo-*D*Fc density (3 σ level) at the A-site (**Figure S4D**). The Fe (II) ion at the A-site, however, could not be refined, probably due to the very low occupancy of the Fe (II) ion at the A-site. In these two crystal structures, B-factors of the Fe (II) ions (47.4 Å^2^/45.5 Å^2^) in protomer B were significantly higher than those of the Fe (II)-ion ligands (40.1 Å^2^/31.8 Å^2^ in average) in these two complex structures (**Table S4**). These facts suggested that the occupancies of the Fe (II) ions are relatively low in these crystals. It would therefore be possible that the alternative conformation could not be clearly observed due to low occupancy of the Fe (II) ion. Of course, we could not exclude the possibility that the Fe (II) shift failed to occur in these crystals for unknown reasons.

In the crystallographic refinement of the DesB-gallate complex (in *P*2_1_) with alternative conformations of the Fe (II) ion, the occupancies of alternative Fe (II) ions were set to 0.5, resulting in the B-factors of 23.6 Å^2^ and 14.6 Å^2^ for the A- and R-sites, respectively (**Table S2**). The average B-factor of the bound gallate, the occupancy of which was set to 1.0, was 28.5 Å^2^. These facts suggest that the occupancies of the Fe (II) ions at the A- and R-sites are different from 0.5. To further analyze the Fe (II) shift found in the *P*2_1_ crystal, we co-crystallized DesB with gallate under anaerobic conditions and determined its crystal structure (**Table 2**). Interestingly, disorder of protomer A and alternative conformations of the Fe (II) ion in protomer B were also observed in the co-crystal structure of the DesB-gallate complex (**Figure S4C**). In addition, B-factors of the Fe (II) ions at the A- and R-sites in the co-crystal structure are similar to those of the DesB-gallate complex (PDB ID: 3WKU) prepared by the soaking method (**Table S2**).

The crystallographic analysis in this study showed alternative conformations of the Fe (II) ion, R- and A-sites, both in *C*222_1_ and *P*2_1_ crystals. Interestingly, no crystal structures showed 100% occupancy of the Fe (II) ion at the A-site. Since B-factor of the bound substrate is comparable to the averaged B-factor of residues around the substrate in each case (**Table S2**), the occupancy of the bound substrate seems to be nearly 1.0. It is therefore unlikely that the partial occupancy of the bound substrate causes the partial occupancy of the Fe (II) ion at the A-site. The observation of the alternative conformation of the Fe (II) ion could be explained by a hypothesis that the shift of the Fe (II) ion is reversible under anaerobic conditions. While the driving force of the large Fe (II) shift remains elusive, the dynamics of the molecule might play an essential role in the shift.

The shift of the Fe (II) ion was further analyzed on the basis of the crystal structures of DesB. Analysis of the van der Waals surface around the active site showed that direct coordination between the hydroxyl groups of gallate and the Fe (II) ion at the R-site cannot be allowed due to steric hindrance (**Figure S5**). For the direct coordination, the Fe (II) ion should be shifted as observed in the DesB-gallate complex. Otherwise there should be significant conformational change around the Fe (II) ion. Since a similar shift of the Fe (II) ion was observed in the related enzyme LigAB, the shift of the Fe (II) ion observed in the DesB-gallate complex would seem to be reasonable. We therefore concluded that the shift of the Fe (II) ion from the R- to A-sites is required for direct coordination between the Fe (II) ion and the substrate.

### Crystal structures of the aerobic DesB-gallate complex

In order to analyze the relationship between the catalytic reaction and the observed Fe (II) shift, we examined whether or not the trapped reaction could be resumed in the crystal under aerobic conditions. First, a crystal of the DesB-gallate complex was prepared under anaerobic conditions. The crystal of the anaerobic DesB-gallate complex was then exposed to an aerobic atmosphere for 30 minutes to resume the catalytic reaction that is blocked under the anaerobic conditions. The aerobic-exposed crystal (hereafter the aerobic substrate complex crystal) was then frozen for diffraction data collection (**Table 1**). The crystal structure of the aerobic substrate complex of DesB showed no bound substrate (**Figure 3C**). The Fe (II) ion was located at the R-site. The occupancy of the Fe (II) ion was set to 1.0 (**Table S2**). The active site structure of the aerobic substrate complex showed no significant differences from that of the substrate-free form. This fact suggests that, upon exposure to aerobic conditions, the catalytic reaction of DesB was resumed in the crystal to cleave the catecholic ring of gallate, and the resultant product was dissociated from the active site. The active site structure of the anaerobic DesB-gallate complex, therefore, appears to represent a productive substrate complex of DesB.

It is worth noting that there is another possible interpretation of this result. It is well known that the catalytic reaction is slow and sometimes inhibited in the crystal due to crystal packing. DesB in the crystal may not catalyze the ring-opening reaction. It is thus possible that the bound substrate in the active site dissociates from the active site and is catalyzed by the DesB enzyme dissolved from the DesB crystal in the solution. In the present setting of the experiment, we cannot exclude this possibility.

### Crystal structure of the DesB-PCA complex

Although DesB cannot catalyze the ring-opening reaction of PCA [6], PCA was expected to bind to the active site due to its structural similarity to gallate. To analyze the reason for the inactivity of DesB for PCA, the crystal structure of the DesB-PCA complex was determined (**Table 1**). As expected, an electron density corresponding to bound PCA was found in the active site of the anaerobic DesB-PCA complex (**Figure 3D**). The position of the bound PCA was nearly the same as that of gallate in the anaerobic DesB-gallate complex. The anomalous difference Fourier density for the Fe (II) ion showed a spherical shape of more than 5 σ at the R-site (**Figure 3D**). Whereas the hydroxyl group OH (4) of PCA is located at nearly the same position as the OH (4) of gallate, the other hydroxyl group OH (3) of PCA forms hydrogen bonds with Glu377’ (**Figure 3D**). These hydrogen bonds correspond to those observed between OH (5) of gallate and Glu377’ in the anaerobic DesB-gallate complex (**Figure 2C**). Since PCA has only two hydroxyl groups, an axial hydroxyl group corresponding to OH (3) of gallate is missing in the DesB-PCA complex. Consequently, PCA cannot induce the Fe (II) shift for the bidentate coordination of the Fe (II) ion, resulting in the inactivity of DesB for PCA. The PCA binding cannot induce formation of a productive complex.

**Figure 4:**
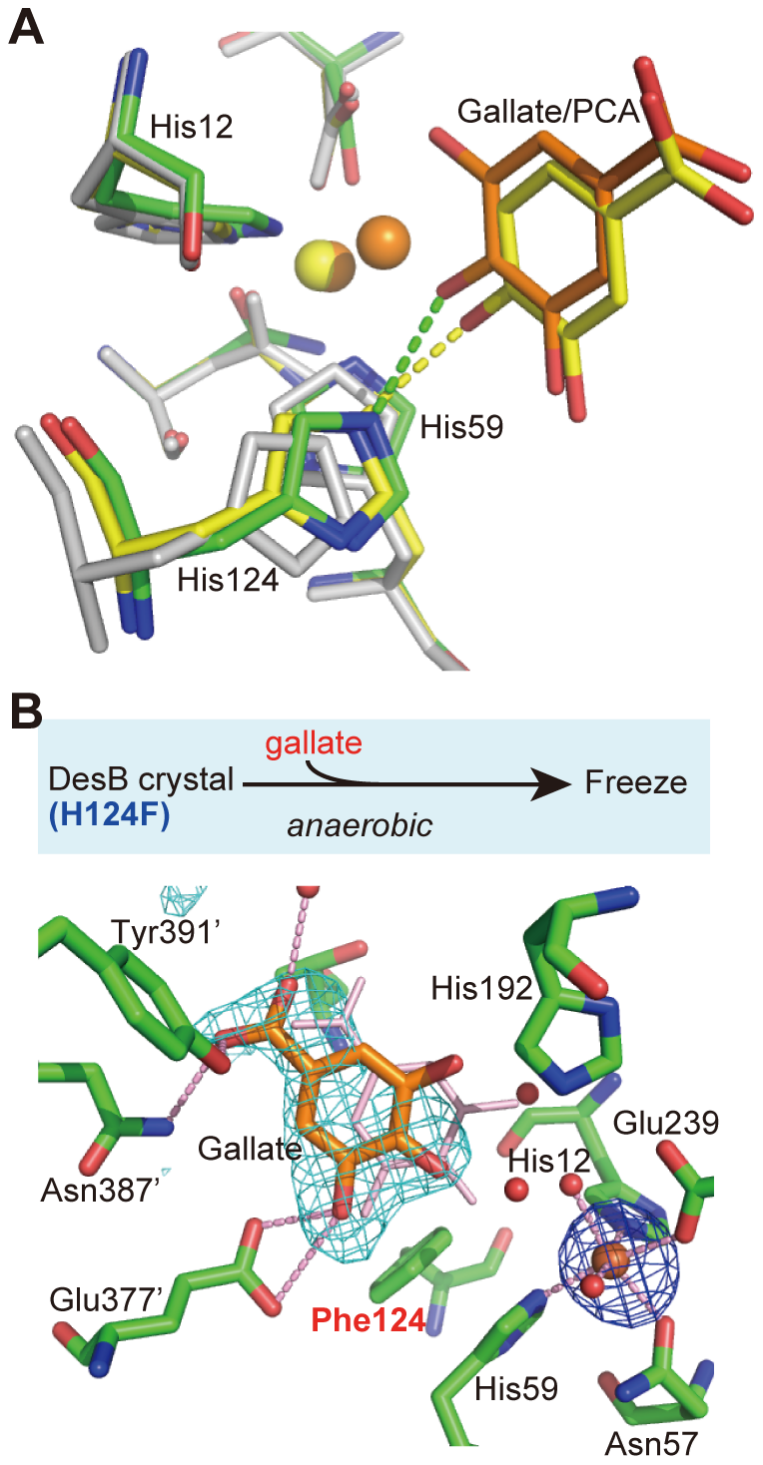
His124 forms a hydrogen bond with a hydroxyl group of gallate and PCA. (**A**) Superposition of the active sites of DesB in the substrate-free (white), anaerobic PCA-complex (carbon atoms in yellow) and anaerobic gallate-complex (carbon atoms in green) forms. Carbon atoms of gallate are shown in orange. (**B**) Crystal preparation scheme of the anaerobic His124Phe DesB-gallate complex (upper panel) and its active site structure with an anomalous difference Fourier map (blue, 5.0 σ) (lower panel). A simulated annealing omit map for gallate (cyan) is also shown (3.0 σ). The thin stick model in pink indicates the position of gallate in the anaerobic DesB-gallate complex.

Although the PCA binding cannot induce an Fe (II) shift, the bound PCA induces a conformational change of His124 as observed in the DesB-gallate complex (**Figure 4A**). In the DesB-PCA complex, His124 significantly shifts towards a hydroxyl group at the equatorial position (OH (4)) and forms a hydrogen bond with the hydroxyl group (**Figure 4A**). Since the aromatic rings of bound PCA and gallate are located at similar positions, the interaction between a hydroxyl group and His124 seems to be critical to properly position the substrate in the active site for the catalytic reaction.

### Crystal structure of the DesB (His124Phe)-gallate complex

The function of His124 was further investigated using the DesB mutant His124Phe, which shows no enzymatic activity for gallate (see above). Crystal structure analysis of the anaerobic DesB (His124Phe)-gallate complex showed that the Fe (II) ion was located at the R-site. Gallate is bound to the active site of the mutant, and the carboxyl group of gallate forms hydrogen bonds with Asn387’ and Tyr391’. However, the location of gallate is different from that found in the wild-type DesB-gallate complex (**Figure 4B**). Since the PCA molecule that forms a hydrogen bond with His124 is located at the productive binding site in the DesB active site (**Figure 4A**), interaction between OH (4) and His124 is required to place gallate at the position, where the hydroxyl groups of gallate coordinate the Fe (II) ion at the A-site to form a productive complex.

## Discussion

**Figure 5:**
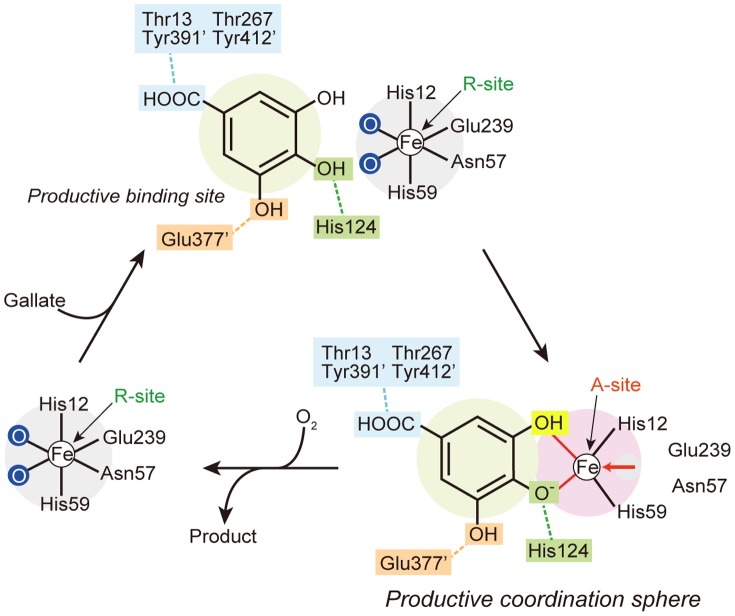
Substrate recognition and Fe (II) shift in the catalytic reaction of DesB. The light green circle represents the productive binding site for gallate. The pink circle represents the productive coordination sphere with the Fe (II) ion at the A-site.

Our crystallographic analyses of DesB showed that gallate is recognized by several hydrogen bonds. These hydrogen bonds can be classified into three groups (**Figure 2C** **,** **Figure 5**). The first group of the hydrogen bonds is formed between DesB and the carboxyl group of gallate. Thr13, Thr267, Tyr391’, and Tyr412’ are involved in this interaction. Since DesB shows only a trace of activity (approximately 1/400 of gallate) for pyrogallol, which lacks the carboxyl group from gallate, the hydrogen bonds of the first group seem to be critical to the substrate binding. The second group of the hydrogen bonds is an interaction between OH (4) of gallate and His124, which seems to be utilized to locate gallate at the productive binding site. The importance of this interaction in the substrate binding is suggested by the structural comparison of the DesB (WT)-gallate, DesB-PCA and DesB (His124Phe)-gallate complexes (**Figures 3D** **,** **Figure 4B**). Gallate cannot be located at the productive binding site without this interaction. The third group of the hydrogen bonds is that between Glu377’ and OH (5) of gallate. Glu377’ seems to determine the substrate arrangement in the active site. As observed in the DesB-PCA complex (**Figure 3D**), Glu377’ interacts with the OH (3) group of PCA and arranges PCA in a flipped manner to form a non-productive complex in the active site. The crystal structure of the DesB-PCA complex suggested that the binding geometry of 3MGA is also regulated by the interaction between Glu377’ and OH (5) of 3MGA (**Figure 1B**), leading to a non-productive binding mode as observed in the DesB-PCA complex. Glu377’ may interact with a hydroxyl group of substrate homologues to stall the catalytic reaction by forming a non-productive complex. It is of note that LigAB has Phe at the corresponding position. Therefore, OH (3) of PCA cannot have a hydrogen bond that flips PCA in the active site of LigAB. As a result, PCA seems to bind to the active site of LigAB with the productive arrangement. Taken together, the three groups of hydrogen bonds in DesB seem to be required to place the substrate at the productive binding site with the productive arrangement. Importantly, the substrate located at the productive binding site with the productive arrangement can induce the shift of the Fe (II) ion. Although gallate and PCA are located at the productive binding site, only gallate can induces the shift of the Fe (II) ion, leading to the direct coordination between the Fe (II) ion and gallate (**Figure 3** **,** **Figure 5**). Since the direct coordination between the Fe (II) ion and two hydroxyl groups of the substrate triggers the catalytic reaction of the extradiol dioxygenases, DesB specifically catalyzes the ring opening reaction of gallate. The separation of the substrate recognition and trigger of the catalytic reaction seems to contribute to the strict substrate specificity of DesB.

The catalytic mechanism of DesB could be predicted on the basis of the present crystallographic and biochemical analyses. The crystal structures of the anaerobic DesB-gallate complexes showed that the relative dispositions of the gallate, His124, and His192 in the DesB-gallate complex are essentially the same as those of PCA, His127b, and His195b in the LigAB-PCA complex, respectively (**Figure 2D**) [9]. A biochemical analysis of MhpB, a related enzyme of DesB and LigAB, has suggested that His115 and His179 serve as catalytic acid and base, respectively [14]. His124 and His192 of DesB, which respectively correspond to His115 and His179 of MhpB, therefore seem to serve as catalytic acid and base. In addition, the structural similarity of the active site among extradiol dioxygenases allows us to predict the O_2_-binding site in DesB (**Figure S6**). The O_2_-binding site of extradiol dioxygenases is located between the catalytic base and the Fe (II) ion [12], [15], [16]. The small cavity at the corresponding position in DesB seems to be the O_2_-binding site of DesB (**Figure S6**).

As described above, the large shift of the Fe (II) ion is one of the unique characters of DesB. While the shift of the Fe (II) ion was also observed in LigAB [9], the coordination sphere was conserved after the shift of the Fe (II) ion. Our crystallographic analysis of DesB, however, showed that the Fe (II) ion at the A-site seems to lose some interactions with the original ligands in the substrate-free form. In particular, Glu239 and Asn57 cannot coordinate the Fe (II) ion at the A-site due to the long distances. However, since the crystallographic analysis in this study was performed at moderate resolution, the precise coordination geometry of the productive complex of DesB remains ambiguous. We are not sure whether or not His12 and His59 coordinate to the Fe (II) ion in the gallate complex. While the details of the productive coordination sphere should be analyzed in the future, we could not exclude the possibility that the productive coordination sphere of DesB is different from the highly conserved His-His-Glu coordination sphere of the extradiol dioxygenases. Interestingly, it has been proposed that the Fe (II) ion in the extradiol dioxygenase serves to organize and electronically connect the catecholic substrate and oxygen, while the potential for electron transfer is tuned by the substrate rather than the protein [27]. Although the interaction between a metal ion and its ligands plays a critical role in regulating the redox potential of the metal ion (s) in typical metal enzymes [28], [29], the Fe (II) ion in the extradiol dioxygenases can be replaced with other metal (s) [6], [27], [30]. This unique character of the metal center of the extradiol dioxygenases may allow a rearrangement of the coordination sphere of DesB in the catalytic reaction.

In this study, we revealed the molecular mechanisms of the substrate binding and the productive complex formation of DesB. Three groups of the hydrogen bonds recognize a substrate and place the substrate in a productive arrangement. In addition, the catalytic reaction of DesB requires the shift of the Fe (II) ion, leading to the bidentate coordination of the substrate to the Fe (II) ion. The formation of the productive coordination sphere then triggers the catalytic reaction. The separation of the substrate recognition and trigger of the catalytic reaction seems to be a key to the strict substrate specificity of DesB. His124 and His192 function as catalytic acid and base for the ring-opening reaction, respectively. To further analyze the molecular mechanisms of the substrate recognition and catalytic reaction of DesB, combination of crystallographic and spectroscopic analyses would be required.

## Supporting Information

Figure S1 **Electron density of DesB.** Stereo view of a 2*m*Fo-*D*Fc map of DesB in the substrate-free form (around the Fe site). Electron densities are contoured at a level of 1 σ ˜..
(PDF)

Figure S2 **CD spectra.** CD spectra of wild-type and His192Phe DesB, which are shown with red and blue lines, respectively. These data suggest that His192Phe DesB retains nearly the same tertiary structure as that of the wild type.
(PDF)

Figure S3 **Structural comparison of the active sites.** The active site structures of (A) DesB and (B) BphC (a type I extradiol dioxygenase) from the *Acidovorax* sp. strain KKS102 in complex with its substrate (2,3-dihydroxybiphenyl: 2,3-DHBP) [11], [12]. Although the relative arrangement of the 2xHis-1xGlu ligands differs between DesB and BphC, the positions of the catalytic His residues (His192 and His194 in DesB and BphC, respectively) are conserved between them. These His residues, which are essential to the enzyme activity, are located adjacent to the axial hydroxyl group of the substrate. In addition, the equatorial hydroxyl group (OH (4) and OH (2) in gallate and 2,3-DHBP, respectively) of each substrate forms a hydrogen bond with a neighbouring residue (His124 and Tyr249 for DesB and BphC, respectively), which is critical to the enzyme reaction.
(PDF)

Figure S4 **The Fe (II) ion shift in the DesB-gallate complex of the** ***P*** **2_1_ crystal. (A)** Alternative conformations of the Fe (II) ion observed in the anaerobic DesB-gallate complex (lower panel) from a *P*2_1_ crystal (PDB ID: 3WKU) (**Table 2**). **(B)** The active site of the DesB-gallate complex without Fe (II) shift (lower panel) in a *P*2_1_ crystal (PDB ID: 3WR4). **(C)** Alternative conformations of the Fe (II) ion observed in the co-crystal of the anaerobic DesB-gallate complex (*P*2_1_) (PDB ID: 3WPM) (**Table 2**). The scheme of crystal preparation is shown in each panel. All Fo-Fc omit maps for the Fe (II) ion are contoured at the 5.0 σ level (blue). (D) Electron density maps of the anaerobic DesB-gallate complex (*P*2_1_) (PDB ID: 3WR4). 2*m*Fo-*D*Fc map is contoured at the 1.5 σ level (pale green). *m*Fo-*D*Fc maps are contoured at 3.0 σ and −3.0 σ levels (red and blue, respectively).
(PDF)

Figure S5 **The Fe (II) ion at the R-site cannot coordinate the gallate.** (**A**) The Fe (II) ion at the A-site can coordinate two hydroxyl groups of the gallate at the reactive position. No steric hindrances occur. (**B**) Since the distance between the Fe (II) and the gallate is too great, the Fe (II) ion at the R-site cannot coordinate the hydroxyl groups of the gallate. The access of the gallate to the Fe (II) ion at the R-site also cannot be allowed due to steric hindrances. All atoms in panels (A) and (B) are shown in van der Waals spheres.
(PDF)

Figure S6 **O_2_-binding site of DesB.** The O_2_-binding site is indicated by a pink dotted ellipsoid. Carbon atoms in His192, which is the catalytic base, are shown in yellow. Carbon atoms of the ligand residues and bound gallate are shown in cyan and orange, respectively. The other carbon atoms are shown in green.
(PDF)

Table S1 **Kinetic parameters of DesB, DesZ, and LigAB [6]** **.**
(PDF)

Table S2 **Summary of occupancies and B-factors for the Fe (II) ion, substrate, and Fe (II) ligands.**
(PDF)

Table S3 **Coordination distances (in Å units).**
(PDF)

Table S4 **Summary of occupancies and B-factors for the Fe (II) ion, substrate, and Fe (II) ligands of the DesB-gallate complex without Fe (II) shift.**
(PDF)
