# Supplementary figures and images for: Molecular Mechanism of Strict Substrate Specificity of an Extradiol Dioxygenase, DesB, Derived from Sphingobium sp. SYK-6
Source: PLoS One. 2014 Mar 21;9(3):e92249. doi: 10.1371/journal.pone.0092249 (PMC3962378; doi:10.1371/journal.pone.0092249)

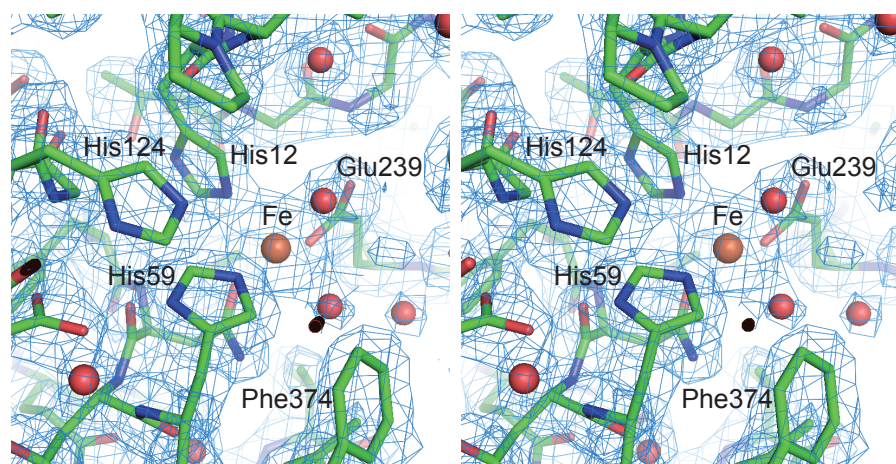

Figure S1

Supplement: Figure S1 — Electron density of DesB. Stereo view of a 2mFo-DFc map of DesB in the substrate-free form (around the Fe site). Electron densities are contoured at a level of 1 σ ˜.. (PDF) [file pone.0092249.s001.pdf]

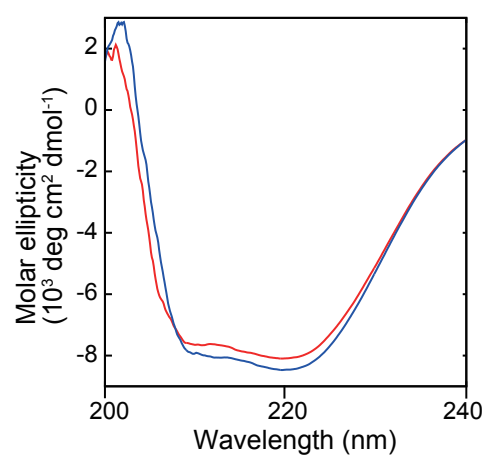

Figure S2

Supplement: Figure S2 — CD spectra. CD spectra of wild-type and His192Phe DesB, which are shown with red and blue lines, respectively. These data suggest that His192Phe DesB retains nearly the same tertiary structure as that of the wild type. (PDF) [file pone.0092249.s002.pdf]

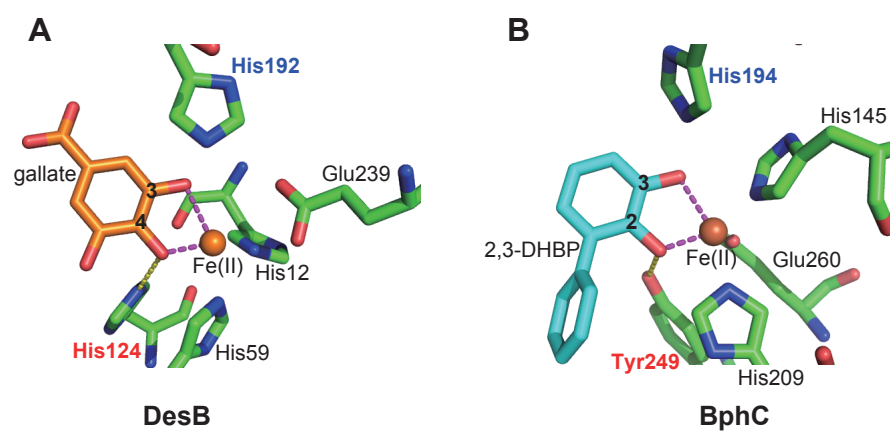

Figure S3

Supplement: Figure S3 — Structural comparison of the active sites. The active site structures of (A) DesB and (B) BphC (a type I extradiol dioxygenase) from the Acidovorax sp. strain KKS102 in complex with its substrate (2,3-dihydroxybiphenyl: 2,3-DHBP) [11], [12]. Although the relative arrangement of the 2xHis-1xGlu ligands differs between DesB and BphC, the positions of the catalytic His residues (His192 and His194 in DesB and BphC, respectively) are conserved between them. These His residues, which are essential to the enzyme activity, are located adjacent to the axial hydroxyl group of the substrate. In addition, the equatorial hydroxyl group (OH (4) and OH (2) in gallate and 2,3-DHBP, respectively) of each substrate forms a hydrogen bond with a neighbouring residue (His124 and Tyr249 for DesB and BphC, respectively), which is critical to the enzyme reaction. (PDF) [file pone.0092249.s003.pdf]

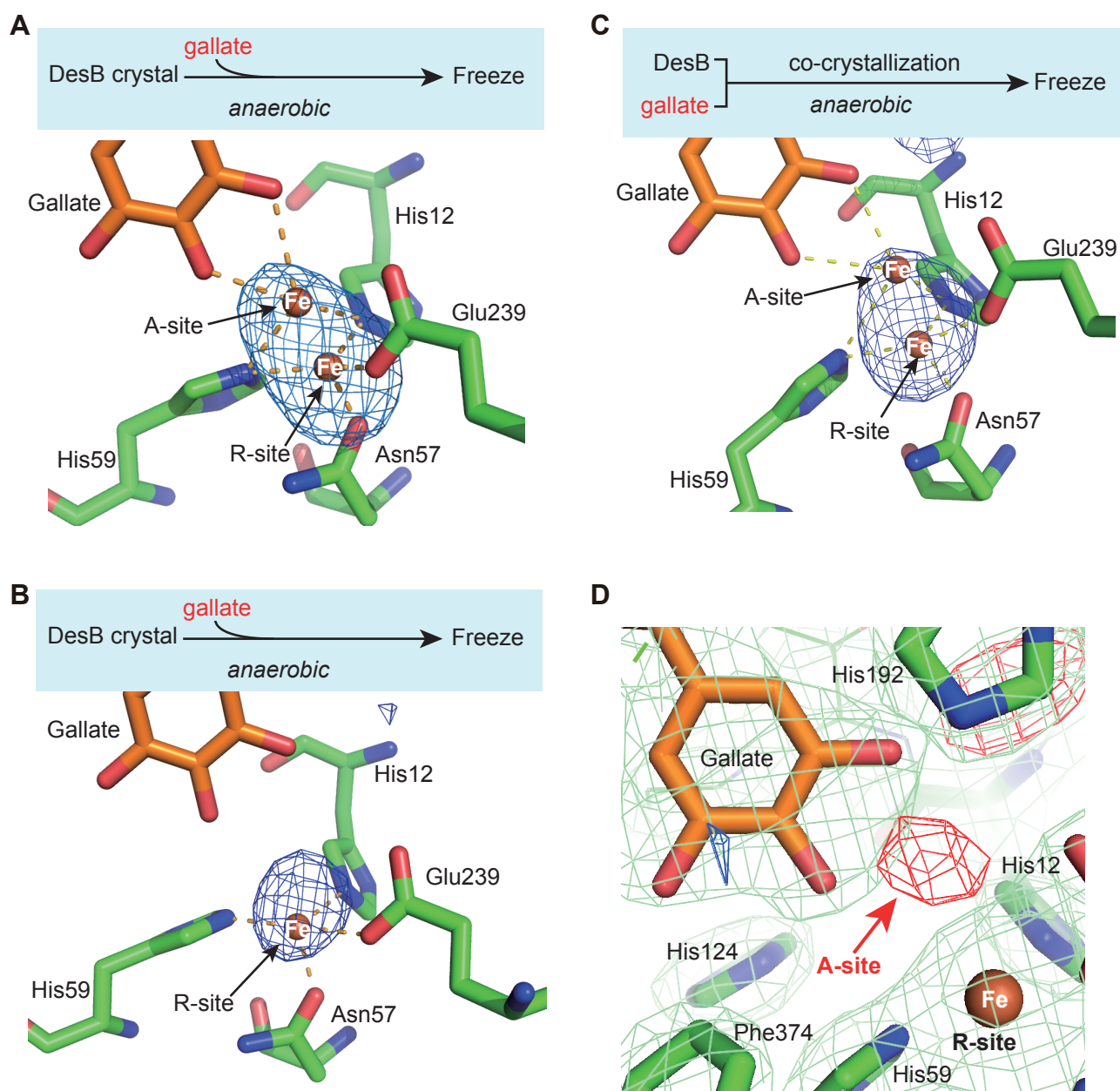

Figure S4

Supplement: Figure S4 — The Fe (II) ion shift in the DesB-gallate complex of the P 21 crystal. (A) Alternative conformations of the Fe (II) ion observed in the anaerobic DesB-gallate complex (lower panel) from a P21 crystal (PDB ID: 3WKU) ( Table 2 ). (B) The active site of the DesB-gallate complex without Fe (II) shift (lower panel) in a P21 crystal (PDB ID: 3WR4). (C) Alternative conformations of the Fe (II) ion observed in the co-crystal of the anaerobic DesB-gallate complex (P21) (PDB ID: 3WPM) ( Table 2 ). The scheme of crystal preparation is shown in each panel. All Fo-Fc omit maps for the Fe (II) ion are contoured at the 5.0 σ level (blue). (D) Electron density maps of the anaerobic DesB-gallate complex (P21) (PDB ID: 3WR4). 2mFo-DFc map is contoured at the 1.5 σ level (pale green). mFo-DFc maps are contoured at 3.0 σ and −3.0 σ levels (red and blue, respectively). (PDF) [file pone.0092249.s004.pdf]

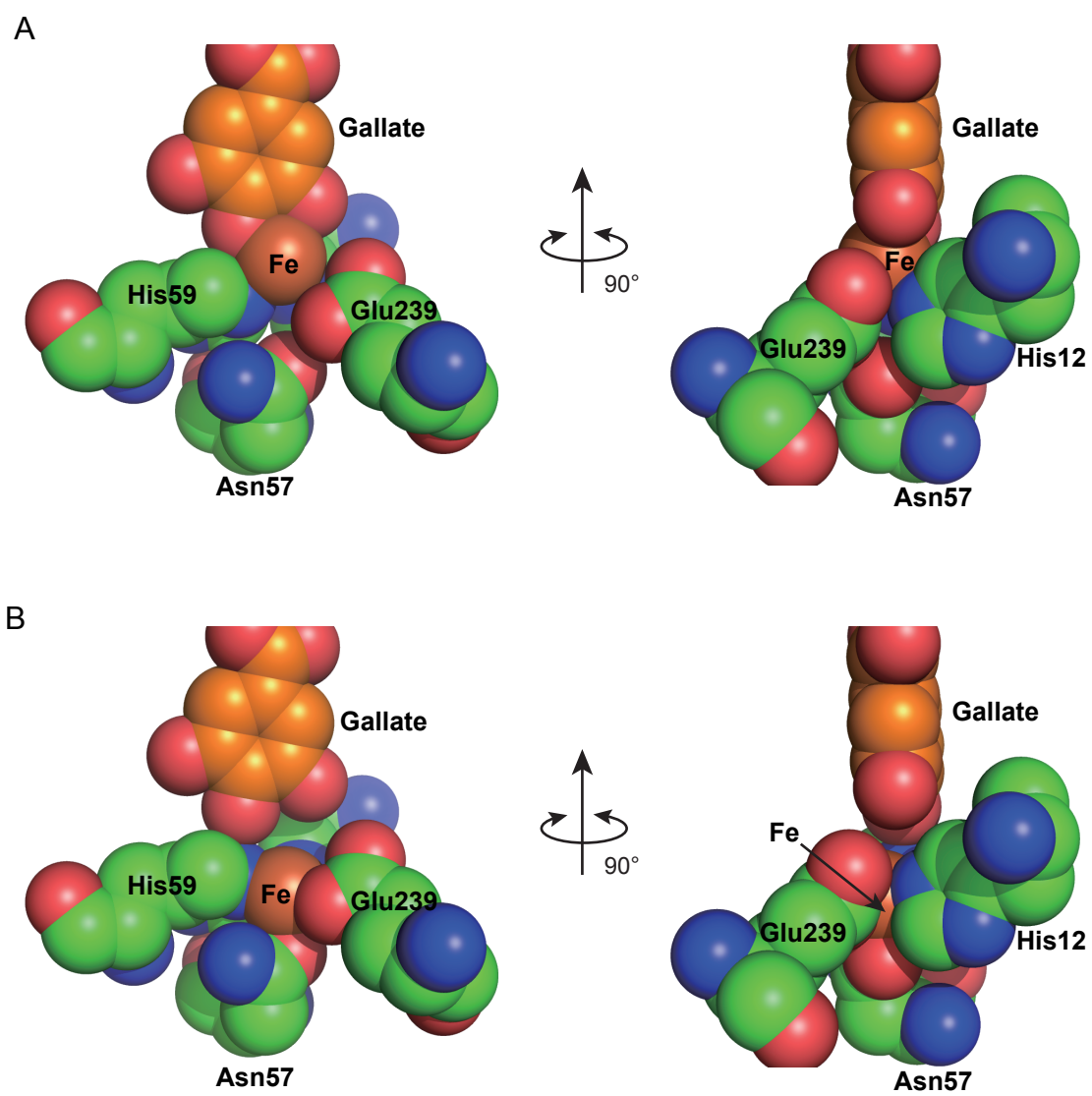

Figure S5

Supplement: Figure S5 — The Fe (II) ion at the R-site cannot coordinate the gallate. (A) The Fe (II) ion at the A-site can coordinate two hydroxyl groups of the gallate at the reactive position. No steric hindrances occur. (B) Since the distance between the Fe (II) and the gallate is too great, the Fe (II) ion at the R-site cannot coordinate the hydroxyl groups of the gallate. The access of the gallate to the Fe (II) ion at the R-site also cannot be allowed due to steric hindrances. All atoms in panels (A) and (B) are shown in van der Waals spheres. (PDF) [file pone.0092249.s005.pdf]

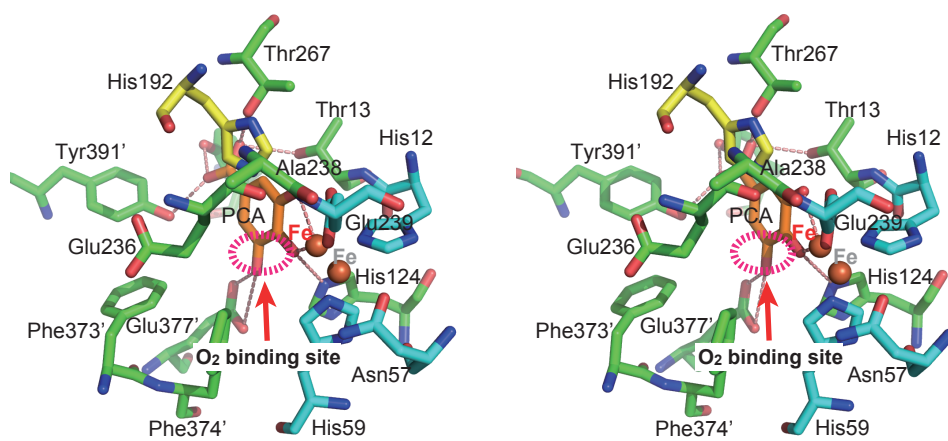

Figure S7

Supplement: Figure S6 — O2-binding site of DesB. The O2-binding site is indicated by a pink dotted ellipsoid. Carbon atoms in His192, which is the catalytic base, are shown in yellow. Carbon atoms of the ligand residues and bound gallate are shown in cyan and orange, respectively. The other carbon atoms are shown in green. (PDF) [file pone.0092249.s006.pdf]
