# Supplementary material for: Molecular Mechanism of Strict Substrate Specificity of an Extradiol Dioxygenase, DesB, Derived from Sphingobium sp. SYK-6
Source: PLoS One. 2014 Mar 21;9(3):e92249. doi: 10.1371/journal.pone.0092249 (PMC3962378; doi:10.1371/journal.pone.0092249)
Supplement: Table S1 — Kinetic parameters of DesB, DesZ, and LigAB [6] . (PDF) [file pone.0092249.s007.pdf]

Table S1. Kinetic parameters of DesB, DesZ and LigAB [6]

Enzyme	k_{cat}/K_m [$M^{-1}sec^{-1}$]		
	Gallate	3MGA	PCA
DesB	4.98×10^5	ND	ND
DesZ	4.50×10^3	1.03×10^4	ND
LigAB	3.19×10^4	7.99×10^2	3.30×10^5
