# Supplementary material for: Molecular Mechanism of Strict Substrate Specificity of an Extradiol Dioxygenase, DesB, Derived from Sphingobium sp. SYK-6
Source: PLoS One. 2014 Mar 21;9(3):e92249. doi: 10.1371/journal.pone.0092249 (PMC3962378; doi:10.1371/journal.pone.0092249)
Supplement: Table S2 — Summary of occupancies and B-factors for the Fe (II) ion, substrate, and Fe (II) ligands. (PDF) [file pone.0092249.s008.pdf]

Table S2. Summary of occupancies and B-factors for the Fe(II) ion, substrate, and Fe(II) ligands.

**DesB**	WT		WT		WT		WT		WT		His124Phe	
Substrate	-		Gallate		Gallate		Gallate		Gallate		Gallate	
Conditions	anaerobic		anaerobic		anaerobic		aerobic		anaerobic		anaerobic	
Space group	$P2_1$		$C222_1$		$P2_1$		$P2_1$		$P2_1$		$P2_1$	
Method	-		soaking		soaking		soaking		co-crystal		soaking	
PDB ID	3WR8		3WR9		3WKU		3WRA		3WPM		3WRB	
**Protomer**	A	B	A	B	A	B	A	B	A	B	A	B
	-	-	-	-	*Disorder*	-	*Disorder*	-	*Disorder*	-	-	-
Occupancy												
Fe(II) A-site	-	-	0.5	0.5	-	0.5	-	-	-	0.5	-	-
Fe(II) R-site	1.0	1.0	0.5	0.5	1.0	0.5	1.0	1.0	1.0	0.5	1.0	1.0
Substrate	-	-	1.0	1.0	-	1.0	-	1.0	-	1.0	1.0	1.0
B-factor (Å^2)												
Overall	22.3	21.9	27.5	29.6	79.0	44.9	38.4	30.8	52.2	32.9	25.0	20.1
Fe(II) A-site	-	-	14.4	18.5	-	23.6	-	-	-	29.7	-	-
Fe(II) R-site	23.0	18.9	17.9	27.8	112.5	14.6	40.5	23.2	55.3	11.0	21.8	13.3
Fe(II) ligands*	18.5	17.5	22.6	28.9	73.1	31.9	32.9	23.9	47.0	21.5	19.3	12.0
Substrate (OH)†	-	-	22.0	31.1	-	31.1	-	-	-	23.0	-	-
Substrate (all) #	-	-	23.1	29.5	-	28.5	-	-	-	23.9	34.0	24.0
Residue around the substrate§			25.9	29.5		33.6				27.9	26.7	15.6

* Fe(II) ligands include His12, Asn57, His59 and Glu239

† Averaged B-factor of two hydroxyl groups that coordinates the Fe(II) ion.

Averaged B-factor of all substrate atoms

§ Averaged B-factor of residues Thr13, His124, His192, Thr267, Glu377’, Tyr391’, and Tyr412’, which are located around the substrate (**Figure 2C**).
