# Supplementary material for: Molecular Mechanism of Strict Substrate Specificity of an Extradiol Dioxygenase, DesB, Derived from Sphingobium sp. SYK-6
Source: PLoS One. 2014 Mar 21;9(3):e92249. doi: 10.1371/journal.pone.0092249 (PMC3962378; doi:10.1371/journal.pone.0092249)
Supplement: Table S3 — Coordination distances (in Å units). (PDF) [file pone.0092249.s009.pdf]

Table S3. Coordination distances (in Å units)

	Active site I†	Active site II†	Average
	**Substrate free form**		
**His12/NE–Fe(II)**	2.18	2.21	2.20
**Asn57/OD–Fe(II)**	2.26	2.31	2.29
**His59/NE–Fe(II)**	2.36	2.28	2.32
**Glu239/OE–Fe(II)**	2.07	2.06	2.07
	**Gallate complex**		
**OH(3)–Fe(II)** §	2.50	2.75	2.63
**OH(4)–Fe(II)** §	1.95	1.91	1.93
**His12/NE–Fe(II)** §	2.54	2.01	2.28
**Asn57/OD–Fe(II)** §	4.33	4.46	4.40
**His59/NE–Fe(II)** §	2.27	2.45	2.36
**Glu239/OE–Fe(II)** §	3.26	3.16	3.21

† Active sites I and II contain protein ligands for the Fe(II) ion from subunits A and B, respectively.

§ Coordination distances in the gallate complex are those between a ligand atom and the Fe(II) ion at the A-site.
