# Supplementary material for: Molecular Mechanism of Strict Substrate Specificity of an Extradiol Dioxygenase, DesB, Derived from Sphingobium sp. SYK-6
Source: PLoS One. 2014 Mar 21;9(3):e92249. doi: 10.1371/journal.pone.0092249 (PMC3962378; doi:10.1371/journal.pone.0092249)
Supplement: Table S4 — Summary of occupancies and B-factors for the Fe (II) ion, substrate, and Fe (II) ligands of the DesB-gallate complex without Fe (II) shift. (PDF) [file pone.0092249.s010.pdf]

Table S4. Summary of occupancies and B-factors for the Fe(II) ion, substrate, and Fe(II) ligands of the DesB-gallate complex without Fe(II) shift.

**DesB**	WT		WT	
Substrate	Gallate		Gallate	
Conditions	anaerobic		anaerobic	
Space group	$P2_1$		$P2_1$	
Method	soaking		soaking	
PDB ID	3WR3		3WR4	
**Protomer**	A	B	A	B
	Disorder	-	*Disorder*	-
Occupancy				
Fe(II) A-site	-	-	-	-
Fe(II) R-site	1.0	1.0	1.0	1.0
Substrate	-	1.0	-	1.0
B-factor (Å^2)				
Overall	70.6	49.7	61.7	42.4
Fe(II) A-site	-	-	-	-
Fe(II) R-site	72.9	47.4	77.5	45.5
Fe(II) ligands*	63.5	40.1	55.4	31.8
Substrate (OH)†	-	44.2	-	32.9
Substrate (all) #	-	41.6	-	34.6
Residue around the substrate§	73.1	45.2	65.3	38.3

* Fe(II) ligands include His12, Asn57, His59 and Glu239
† Averaged B-factor of two hydroxyl groups that coordinates the Fe(II) ion.
# Averaged B-factor of all substrate atoms
§ Averaged B-factor of residues Thr13, His124, His192, Thr267, Glu377', Tyr391', and Tyr412', which are located around the substrate (**Figure 2C**).
